# Surprising Threats Accelerate Conscious Perception

**DOI:** 10.3389/fnbeh.2022.797119

**Published:** 2022-05-13

**Authors:** Jessica McFadyen, Naotsugu Tsuchiya, Jason B. Mattingley, Marta I. Garrido

**Affiliations:** ^1^Queensland Brain Institute, University of Queensland, Brisbane, QLD, Australia; ^2^Max Planck UCL Centre for Computational Psychiatry and Ageing Research, University College London, London, United Kingdom; ^3^Australian Research Council Centre of Excellence for Integrative Brain Function, Clayton, VIC, Australia; ^4^School of Psychological Sciences and Turner Institute for Brain and Mental Health, Monash University, Clayton, VIC, Australia; ^5^Center for Information and Neural Networks (CiNet), National Institute of Information and Communications Technology (NICT), Osaka, Japan; ^6^Advanced Telecommunications Research Computational Neuroscience Laboratories, Kyoto, Japan; ^7^School of Psychology, University of Queensland, Brisbane, QLD, Australia; ^8^Canadian Institute for Advanced Research, Toronto, ON, Canada; ^9^Melbourne School of Psychological Sciences, The University of Melbourne, Melbourne, VIC, Australia

**Keywords:** consciousness, perception, faces, breaking continuous flash suppression, eeg, expectation, emotion

## Abstract

The folk psychological notion that “we see what we expect to see” is supported by evidence that we become consciously aware of visual stimuli that match our prior expectations more quickly than stimuli that violate our expectations. Similarly, “we see what we want to see,” such that more biologically-relevant stimuli are also prioritised for conscious perception. How, then, is perception shaped by biologically-relevant stimuli that we did not expect? Here, we conducted two experiments using breaking continuous flash suppression (bCFS) to investigate how prior expectations modulated response times to neutral and fearful faces. In both experiments, we found that prior expectations for neutral faces hastened responses, whereas the opposite was true for fearful faces. This interaction between emotional expression and prior expectations was driven predominantly by participants with higher trait anxiety. Electroencephalography (EEG) data collected in Experiment 2 revealed an interaction evident in the earliest stages of sensory encoding, suggesting prediction errors expedite sensory encoding of fearful faces. These findings support a survival hypothesis, where biologically-relevant fearful stimuli are prioritised for conscious access even more so when unexpected, especially for people with high trait anxiety.

## Introduction

The ability to predict, detect, and make decisions about danger is essential for survival. In humans, threatening visual stimuli are detected more quickly and are more difficult to disengage from than non-threatening stimuli (Smith and Lane, 2016). Danger, however, is not always clearly visible. We must also be able to detect potential threats in visually ambiguous situations, such as when observing from a distance, under low light conditions, or when hunted by a camouflaged predator (Parasuraman and Galster, 2013). Threatening and other emotionally-salient stimuli are, indeed, more consciously accessible than neutral stimuli under difficult viewing conditions (Ohman, 2005; Straube et al., 2006; Vieira et al., 2017). At the same time, however, conscious perception of ambiguous visual stimuli is highly susceptible to the influence of prior expectations, such that we tend to see what we expect to see Hohwy et al. (2008) and Pinto et al. (2015). How, then, do these two neural processes interact when we are faced with a threat we did not expect?

Predictive coding theory suggests that conscious perception is the result of a constant stream of hypothesis testing, whereby sensory evidence and prior expectations are integrated to form a posterior probability distribution (Rao and Ballard, 1999; Friston and Kiebel, 2009). This framework accounts for empirical evidence showing that, when sensory input is imprecise, prior expectations are more likely to bias conscious experience (Panichello et al., 2012). For example, motion perception is biased towards prior expectations when motion is less coherent and more ambiguous (Hesselmann et al., 2010; Vetter et al., 2014). Similarly, when two different stimuli are simultaneously presented to each eye using dichoptic presentation (e.g., a stereoscopic mirror set-up or prism lenses), conscious perception is more stable for (and switches more rapidly to) more predictable stimuli (Hohwy et al., 2008; Pinto et al., 2015; Meijs et al., 2018). Expectations themselves can be established explicitly, for instance by a cue preceding a stimulus (Costello et al., 2009; Chang et al., 2015; Pinto et al., 2015; Meijs et al., 2018), or implicitly, such as by how frequently a stimulus has occurred (Aru et al., 2016; Barbosa et al., 2017; Gordon et al., 2017).

Previous studies have shown that prior expectations bias or “sharpen” sensory representations in the visual cortex (Kok et al., 2012, 2013, 2017; Summerfield and de Lange, 2014; Gandolfo and Downing, 2019). Moreover, neural activity measured at the scalp is enhanced for stimuli matching prior expectations during early processing (100 ms post-stimulus onset; Kouider et al., 2015) but is thereafter boosted for surprising stimuli in event-related potential (ERP) components such as the pre-attentive “mismatch negativity” from approximately 100–250 ms post-stimulus onset (Garrido et al., 2009), as well as later slow waves (Kouider et al., 2015) such as the P300 component (Polich, 2007) or decision-related central-parietal positivity (CPP; (Rungratsameetaweemana et al., 2018b; van den Brink et al., 2021).

Like predictable stimuli, threatening stimuli are also prioritised for conscious access (Singer et al., 2012; Schmack et al., 2016; Otten et al., 2017). This can be assessed by breaking continuous flash suppression (bCFS), a technique in which participants become increasingly aware of a stimulus presented to one eye as the contrast of a competing stimulus presented to the other eye is gradually decreased (Tsuchiya and Koch, 2005; Jiang et al., 2007). Fearful faces, snakes, spiders, and fear-conditioned stimuli all tend to be consciously perceived earlier than neutral stimuli during bCFS (Gayet et al., 2016; Gomes et al., 2017). Fearful stimuli have also been shown to increase the rate of evidence accumulation even when unconsciously presented (Tipples, 2015; Lufityanto et al., 2016). There has, however, been little investigation into how the prioritisation of fearful stimuli for conscious access is influenced by prior expectations.

We propose three hypotheses for how prior expectations might influence conscious access to suppressed threatening and neutral stimuli. The first is the **Emotional Exaggeration Hypothesis**, which proposes that an effect of expectation on conscious perception is exaggerated for emotional stimuli (i.e., we “see what we expect to see” even more so if what we expect is dangerous). This may arise from amplification of affective sensory processing (Cornwell et al., 2017) via modulatory connections from amygdala to primary sensory cortices (Aizenberg et al., 2019; Chen et al., 2014), or a gain in amplitude due to increased precision of affective priors (Otten et al., 2017). In support of this hypothesis, previous studies have found neural activity evoked by surprise is larger and earlier for affective than neutral stimuli (Vogel et al., 2015; Chen et al., 2017; Kovarski et al., 2017).

As an alternative to the Exaggeration Hypothesis, we consider the **Survival Hypothesis**, where threat negates or reverses an effect of expectation on conscious perception. This captures the notion that, even in situations where a threat is unexpected, it is still vital (perhaps even more vital) that we can rapidly respond (Ouden et al., 2012). A response gain mechanism for affective stimuli would presumably be at play in both the Emotional Exaggeration and Survival hypotheses. Additionally, however, the survival hypothesis postulates that prediction errors for affective stimuli might be subserved by specific neural architecture that facilitates more rapid updating of conscious content. Subcortical “survival circuits” involving the brainstem, thalamus, and amygdala facilitate unconscious affective processing (Morris et al., 1999; Liddell et al., 2005; Ohman et al., 2007; Tamietto and de Gelder, 2010; McFadyen et al., 2019) and thus might circumvent or otherwise interact with the influence of top-down expectations, resulting in earlier conscious access to emotional stimuli, hastened by surprise (Hohwy, 2012). In support of the Survival Hypothesis, previous research has shown unexpected threats are more frequently detected than unexpected neutral images and evoke stronger physiological responses, even under high perceptual load (Wiemer et al., 2013; New et al., 2015; Gao and Jia, 2017). Additionally, threat cues have been shown to enhance perceptual discrimination of subsequent threat stimuli (Sussman et al., 2017; Imbriano et al., 2020).

A third possibility is that threat and expectation do not interact at all. For example, inattentional blindness research has found no advantage of unexpected threats versus non-threats for entering awareness (Calvillo and Hawkins, 2016; Beanland et al., 2018). Hence, we also considered the **Additive Hypothesis**, which is that both expectation and emotional content independently accelerate conscious perception without interacting.

To test the three hypotheses above, we conducted two bCFS experiments. In each experiment, participants were presented a series of faces with either neutral or fearful expressions. Faces were presented in blocks where one emotional expression occurred more frequently (5:6) than the other, thus inducing a contextual expectation for the more frequently presented emotional expression in a block (Kiebel et al., 2008). Participants performed an orthogonal discrimination task (i.e., whether faces were rotated clockwise or counterclockwise), from which we measured response times as an indicator of perceptual decision-making speed. In both experiments, we examined whether response times differed as a function of emotional expression and/or prior expectations. Experiment 1 served as an exploratory behavioural investigation, and Experiment 2 incorporated electroencephalography (EEG) to determine whether the time course of underlying neural activity differed between neutral and fearful faces as a function of expectation.

## Materials and Methods

### Participants

We recruited participants through the University of Queensland’s Participation Scheme, which draws from adults within the local community. A target sample size of *N* = 30 was derived from previous studies using affective bCFS paradigms (Stein et al., 2012; Gayet et al., 2016). Experiment 1 consisted of 31 participants (14 male, 17 female) aged between 18 and 33 years (*M* = 22, SD = 3). Experiment 2 consisted of 33 participants (18 male, 15 female) aged between 18 and 28 years (*M* = 22, SD = 2). For Experiment 2, one subject was excluded from all analyses for failing to respond on too many trials (41.43%) and another was excluded from EEG (but not behavioural) analyses due to a technical error with EEG triggers. This left a sample of 32 participants (18 male, 14 female, mean age = 22) included in behavioural analyses and a sample of 31 participants (17 male, 14 female, mean age = 22) included in EEG analyses. All participants reported having normal vision without the need for glasses or contact lenses. Participants were compensated AUD$20 per hour for their time and provided written consent. This study was approved by the University of Queensland’s Human Research Ethics Committee.

### Stimuli

We collected face stimuli from a variety of experimentally-validated databases to maximise the number of unique face stimuli presented, thus minimising any effects of visual familiarity to better isolate the effect of the emotional expression itself. Our set included 24 images from the Amsterdam Dynamic Facial Expressions Set (ADFES; Van der Schalk et al., 2011), 132 images from the Karolinska Directed Emotional Faces set (KDEF; Lundqvist et al., 1998), 52 images from the NimStim set (Tottenham et al., 2009), and 58 images from the Warsaw Set of Emotional Facial Expression Pictures (WSEFEP; Olszanowski et al., 2015). The final selection consisted of 266 images of Caucasian adults (66 females and 67 males) displaying either a neutral or fearful facial expression.

**FIGURE 1:**
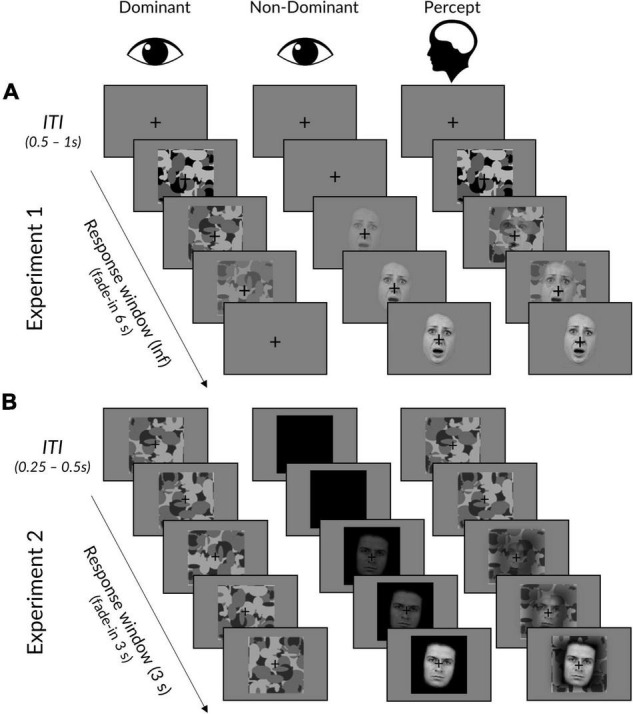
Interocular suppression paradigm for Experiments 1 and 2. **(A)** In Experiment 1, face stimuli linearly increased from 0 to 100% contrast over 6 s in the dominant eye, while the mask did the opposite in the dominant eye. Trials ended upon response, followed by an ITI displaying a fixation cross. **(B)** Experiment 2 was the same, except that: (i) face stimuli linearly increased over a period of 3 s to a titrated subject-specific maximum contrast, (ii) masks remained at maximum contrast, (iii) face background was black rather than grey, (iv) trials ended after 3 s regardless of whether a response had been made, and (v) masks remained throughout the ITI. The “Percept” column illustrates a typical subjective experience, in which initially only a mask could be perceived, followed by a mixed perception of both mask and face. Facial stimuli reproduced with permission from the Warsaw Set of Emotional Facial Expression Pictures (WSEFEP), available at http://www.emotional-face.org.

We cropped the hair, neck, and shoulders from all face stimuli (see Figure 1). We then centred the faces within a 365 x 365 pixel square with a grey background for Experiment 1 and a black background for Experiment 2 (to increase the contrast and maximise the visually-evoked EEG response to faces). We normalised luminance and contrast across all images using the SHINE toolbox (Willenbockel et al., 2010), such that neither mean luminance (i.e., greyscale pixel value) nor contrast (i.e., standard deviation of luminance) differed significantly between neutral and fearful faces (luminance: neutral = 125.080, fearful = 124.681, *t*(130) = 1.954, *p* = 0.106; contrast: neutral = 125.903, fearful = 125.472; *t*(130) = 2.038, *p* = 0.088; Bonferroni-corrected for two comparisons).

We used Mondrian images made using code available online (http://martin-hebart.de/webpages/code/stimuli.html; as used by Stein et al., 2014) to mask the stimuli during rivalry (see Figure 1). The Mondrian images were presented at 125% of the size of the face stimuli to ensure that faces were sufficiently masked (luminance = 127.894, contrast = 127.680).

### Procedure

#### Dichoptic Presentation

After completing the consent form, participants completed the self-report 40-item State-Trait Anxiety Inventory (STAI; Spielberger et al., 1971). We then determined the participants’ ocular dominance using the Miles Test (Miles, 1930). Participants then sat approximately 1.1 m (Experiment 1) or 0.55 m (Experiment 2) from a 22” LCD monitor (1980 × 1020 resolution) with a black screen divider placed between the participant and the monitor. For Experiment 1, each participant positioned their head in a chin and head rest, to which prism lenses (12 prism diopters, base out) were attached and secured with a foam strap. For Experiment 2, stereoscopic mirrors were used instead of prism lenses.

In both experiments, participants completed a short calibration task and the apparatus was adjusted (i.e., angle of mirrors/prism lenses, computer monitor height, etc.) to ensure that the stimuli presented to each eye were perceived to be in the same location in space (i.e., completely overlapping in the centre of field of vision) and that only one stimulus could be perceived with each eye. An eye tracker was also used to ensure that participants did not close one eye during the experiment (which would interrupt the interocular suppression).

#### Behavioural Task

Each trial began with the mask presented at 100% contrast to the participant’s dominant eye and a face stimulus presented at 0% to the other eye (see Figure 1). In Experiment 1, the face stimulus contrast would linearly ramp up to 100% contrast over a period of 6 s, while the mask simultaneously ramped down to 0% contrast. Experiment 2 was the same, except that the time period was reduced to 3 s (to reduce experiment length and increase the number of trials) and the mask contrast was fixed at 100% (to avoid an onset effect in the EEG signal).

In both experiments, face stimuli were pseudo-randomly rotated 5° clockwise or counter-clockwise. Participants were instructed to click the left (for faces rotated counterclockwise) or right (for faces rotated clockwise) mouse button as soon as they could perceive the face and judge its orientation. Participants were told to prioritise response accuracy, but to also respond as fast as possible so that their response time occurred as close as possible to the moment of subjective awareness. In Experiment 1, trials ended upon response (if responses were over 6 s, the face remained at 100% and the mask at 0% until response), whereas in Experiment 2, trials always ended after 3 s regardless of response. Between trials, a fixation cross was presented at the centre of each left and right image frame. The duration of the inter-trial interval (ITI) jittered randomly between 0.5 and 1 s at a step of 0.1 s for Experiment 1, and between 0.25 and 0.50 s at a step of 0.05 s for Experiment 2.

There were 8 blocks in Experiment 1 and 14 blocks in Experiment 2. In both experiments, participants were informed that some blocks would contain more of one emotional expression than others but that this was irrelevant to their task. Half the blocks contained predominantly (83%) neutral faces while the other half contained predominantly fearful faces. The dominant emotional expression was indicated at the beginning of each block by a 5-s presentation of the word “neutral” or “fearful.” Neutral and fearful blocks were alternated, with the starting block emotion counterbalanced across participants. There were 90 trials per block and each block began with at least two trials of the predominant emotion. The presentations of rare and unexpected (17%) emotional faces were thereafter spaced apart by 2–7 trials, following a Gaussian distribution. There were 720 total trials for Experiment 1 (300 expected and 60 unexpected trials per neutral/fearful expression) and 1,260 total trials for Experiment 2 (525 expected and 105 unexpected trials per neutral/fearful expression).

#### Titration Procedure

In Experiment 2, participants completed a titration task while the EEG cap was set up. The purpose of the titration was to adjust the relative contrast between the face and the mask so that all participants could make an accurate response (and thus could consciously perceive the face) before the end of each trial. The goal response time was 2 s (i.e., two thirds of the way into the trial).

The titration task consisted of four blocks: two neutral-dominant and two fearful-dominant blocks in an alternate order, with the starting block counterbalanced across participants. Each block contained 90 trials, with 83% dominant emotion presentations and 17% rare emotion presentations.

The titration trials were the same as the trials in Experiment 2 (see Figure 1), except that the titration began with the mask invisible (0% contrast) and the face fully visible (100% contrast). Using the Palamedes toolbox (Prins and Kingdom, 2009), the relative contrast between the mask and the face was adjusted per trial, such that if the response was faster than 2 s, the face contrast in the next trial would decrease and mask contrast would increase (the sum of contrasts was always 100%), and vice versa for responses slower than 2 s. The stepwise function used for these trial-by-trial adjustments began with 10% contrast adjustments, which were reduced by 2% each time a reversal (i.e., a change in response type; fast to slow, or slow to fast) was made. After four reversals, contrast adjustments were fixed at 2%.

These staircases were constructed independently for the first two blocks, one of which was neutral-dominant and the other fearful-dominant. The final contrast value pair from each block type was then used as the starting point for the next block of each dominant emotion, giving a fine-tuned contrast set built across two blocks of 90 trials each per neutral and fearful block type. The neutral-dominant and fearful-dominant contrast sets were then averaged together to produce a face contrast value per participant. These ranged from 53.23 to 91.68% (*M* = 76.75%, SD = 10.25%) across participants (mask contrast values were equal to 100% minus the face contrast). Each participant’s final titrated face contrast value was used as the maximum contrast for all face stimuli (neutral or fearful, in any block type) presented in the main experiment.

### Behavioural Analysis

#### Multilevel Modelling

For both Experiments 1 and 2, we analysed differences in response times between conditions using linear mixed effects modelling using the “lme4” package (Bates et al., 2015) in R v3.4.3 (R Core Team). This approach encapsulates single-trial data across all participants by modelling both random effects (e.g., each subject) and fixed effects (e.g., the conditions of interest: emotion and expectation). For both Experiments 1 and 2, our model of response time consisted of a random effect of subject, plus an interaction between emotion and expectation (model formula: RT ∼ Emotion × Expectation + (1 | Subject)). We determined the significance of each fixed effect’s *t*-statistic using the “lmerTest” package, which recruits a Satterthwaite’s degrees of freedom method (Kuznetsova et al., 2017). We also determined the overall significance of the modelled fixed effects by conducting a χ^2^ test between the model and a null model only including the random effect.

To investigate the influence of individual differences in trait anxiety on response time, we pooled the data from Experiments 1 and 2, z-scoring the response times within each experiment to account for the different trial lengths. We then added each participant’s mean-centred trait anxiety score as a third interaction term (model formula: RT ∼ Emotion × Expectation × Anxiety + (1 | Subject) and compared this model against one without anxiety included using a χ^2^ test. Questionnaire scores were missing for one subject in Experiment 1, which was thus excluded from the analysis.

For all models, we computed the variance inflation factor (VIF) using the “car” (Companion to Applied Regression) package (Fox and Weisberg, 2018) to ensure that effects of multicollinearity were minimal (VIF threshold = 4). All models were constructed using data that excluded incorrect responses or responses that were faster than 500 ms (indicating an accidental button press). We then also excluded any trials that were more than 3 standard deviations from each subject’s mean response time. To help equate the number of expected and unexpected trials, we selected only the expected trials that immediately preceded each unexpected trial (i.e., the last standard in each chain of standards). Across subjects, there were an average of 58 trials per condition in Experiment 1 (range = 45–60) and an average of 98 trials per condition in Experiment 2 (range = 49–105).

Bayesian analyses were conducted in JASP v0.16 (JASP Team, 2021) with a default Cauchy prior width of 0.707.

#### Drift Diffusion Modelling

We conducted drift diffusion modelling to investigate whether response times in the orientation discrimination task were influenced by parameters directly pertaining to decision-making (drift rate and decision boundary) versus other processes (i.e., non-decision time). We modelled response times using the EZ-Diffusion Model (Wagenmakers et al., 2007), a simplified form of the Drift Diffusion Model (Ratcliff, 1978) for two-alternative forced choice tasks. The EZ-Diffusion Model is well suited to the current study, as it is designed for sparse data that contain fewer error trials (here, participants performed the orientation discrimination task with very high accuracy; Experiment 1: *M* = 97.30%, SD = 1.44%; Experiment 2: *M* = 93.38%, SD = 2.18%), and assumes a starting point for decision-making that is equidistant between each decision boundary (here, face orientation was random across trials, and thus could not be anticipated prior to trial onset; (Wagenmakers et al., 2007; van Ravenzwaaij et al., 2017).

For each participant and for each condition, we computed the mean response time, response time variance, and mean accuracy (i.e., proportion correct) across all trials (including all standards, to maximise trial numbers). As the EZ-Diffusion Model requires at least one incorrect and one correct trial, we excluded 24 out of 63 subjects who scored 100% accuracy in any condition. We fixed the scaling parameter, *s*, to 0.1, in line with previous research (Wagenmakers et al., 2007). We then estimated parameters *v* (drift rate), *b* (decision threshold), and *T*_*er*_ (non-decision time) per subject, per condition. To estimate the effect of emotion and expectation on each parameter, we z-scored each subject’s parameter estimates and then conducted three separate 2 x 2 repeated-measures ANOVAs with experiment (1 or 2) as a between-subjects factor.

### Electroencephalography

#### Electroencephalography Acquisition

Neural activity was continuously recorded using a BioSemi Active Two 64 Ag-AgCl electrode system (BioSemi, Amsterdam, Netherlands). Participants were fitted with a nylon cap containing 64 Ag/AgCl scalp electrodes positioned according to the international 10–20 system. Continuous data were recorded using BioSemi ActiView software (BioSemi, 2007), filtered online (0.01–208 Hz amplifier band pass filter), and then were digitised and stored at a sampling rate of 1024 Hz with 24-bit A/D conversion. We measured horizontal and vertical electrooculograph (EOG) signals with flat bipolar Ag/AgCl electrodes. The experiment was conducted in an electrically-shielded Faraday cage to minimise noise and all data was recorded with electrode impedance levels under 25 kΩ.

#### Electroencephalography Preprocessing

We preprocessed the EEG data using SPM12 (Wellcome Centre for Human Neuroimaging), OSL (the OHBA Software Library, from OHBA Analysis Group), and custom code written in MATLAB R2018b (MathWorks). First, the continuous data were imported into SPM12 and an average reference was applied. A bandpass filter from 0.1 to 45 Hz was then applied alongside a 50 Hz notch filter for line noise. The data were then downsampled to 100 Hz and automatically denoised using AFRICA (ArteFact Rejection using Independent Component Analysis) in OSL (OHBA Analysis Group). This procedure uses independent components analysis to decompose the data into independent spatiotemporal components. These components were then automatically classified as artifactual by using the combined spatial topography, time course, time course kurtosis, and frequency spectrum of all components (e.g., eye blink artefacts exhibited high kurtosis (>20), a repeated pattern in the time course, and consistent spatial topographies). The number of excluded components was limited to a maximum of 20 and were rejected by subtracting them out of the data. Electrodes and trials with significant outlier activity were automatically identified using a generalised extreme studentized deviate test (α = 0.05).

The denoised data were then segmented into −0.1 to 3-s epochs time-locked to the beginning of each trial (i.e., when a face stimulus contrast was 0% and began to increase in contrast from the next frame). Bad electrodes were interpolated using a weighted neighbour approach in Fieldtrip (Oostenveld et al., 2011). The flashing Mondrian mask induced a 10 Hz frequency in the data (plus its harmonics), and so we smoothed all epochs using a moving average with a 200 ms sliding window. Epochs with extreme amplitude were excluded using GESD (α = 0.05). A set of response-locked epochs were then created by shifting the time axis so that time zero coincided with response onset. All epochs were baseline-corrected using a −50 to 0 ms window pre-trial onset.

#### Electroencephalography Analysis

##### Cluster-Based Permutation

We used nonparametric cluster-permutation testing implemented in Fieldtrip to statistically test differences in neural amplitude between our conditions whilst correcting for multiple comparisons (Maris and Oostenveld, 2007). We adopted a two-level approach, with the first level constituting trial-by-trial effects for individual subjects and the second level constituting the consistency of these effects across the group.

At the first level, we excluded any trials with incorrect responses, artefactual data, or where response times were faster than 500 ms (indicating an accidental button press). We then excluded any trials that were more than 4 standard deviations from the participant’s mean response time. We compared neutral with fearful face trials, as well as expected with unexpected trials. To compute an interaction between emotion and expectation on neural activity, we subtracted the average response to expected faces from single trial responses to unexpected faces, separately for each emotional expression (i.e., unexpected neutral faces minus an average response to expected neutral faces, and unexpected fearful faces minus an average response to expected fearful faces; similar to Lieder et al., 2013). We then compared the resultant difference waves between neutral and fearful expressions.

We conducted a series of two-tailed *t*-tests between each condition of interest using cluster-based permutation (100 permutations). This produced maps of multiple-comparison-corrected *t*-statistics per subject, per contrast (i.e., neutral vs fearful, expected vs unexpected, fearful and neutral mismatch). We brought these individual-subject *t*-maps to the second level, where we conducted two-tailed one-sample cluster-based permutation *t*-tests on each set (500 permutations). In follow-up testing, we also added a regressor for each subject’s mean-centred trait anxiety score.

To investigate a correlation between response time and drift-diffusion model parameters, we regressed each subject’s *t*-maps produced by one-sample *t*-tests per condition across all trials, and then performed a correlation analysis at the group level. Note that all *p* values reported from cluster-based permutation analyses are family-wise-error-corrected at α = 0.05.

##### ERP Change Points

Although cluster-based permutation testing provides a powerful means for testing for broad differences in neural activity between conditions (while correcting for family-wise error), the cluster-forming procedure does not allow inferences at the level of specific electrodes or time-points (Sassenhagen and Draschkow, 2019; Fields and Kuperberg, 2020). Thus, in order to investigate differences in the latency of neural activity between conditions, we derived a method for detecting changes in neural activity at a finer scale. We were specifically interested in the earliest detectable difference in neural activity between conditions prior to response, rather than during conscious viewing post-response. As such, the following method entailed a trial-by-trial detection of transient changes in neural activity preceding response onset.

To identify transient changes in neural activity per trial, per electrode, we used a change point detection procedure implemented in the “findchangepts” MATLAB function. This function automatically identifies abrupt changes in a vector by assessing the point at which there is the most significant change in linear slope (Lavielle, 2005; Killick et al., 2012). We applied the “findchangepts” function to each trial and each electrode using an expanding window approach. In this approach, the function was applied to multiple time windows that each began from 0.100 s post-trial onset (to reduce implausibly fast onsets when face stimuli were almost completely transparent) and extended up to 0.3 preceding response onset (to minimise noise related to preparatory motor activity) in steps of 50 ms. As such, earlier samples were included in all windows, while later samples were included only in longer windows. Thus, by applying the “findchangepts” function to each window, the resultant vector of change points would overrepresent earlier changes, as these shorter windows were uninfluenced by later significant changes (e.g., motor preparation/execution).

We identified “early” change points in neural activity as the most frequent change point identified across all incrementally-increasing windows. We then repeated the process but in reverse, where windows incrementally increased from response onset back to stimulus-onset. Thus, the most frequent value in the resultant vector indicated “late” change points.

## Results

### Behaviour

#### Expectations Differentially Modulate Responses to Emotional Faces

In Experiment 1, our aim was to investigate whether neutral and fearful faces modulated response times differently depending on whether they were expected or not. We entered single-trial response time data into a linear mixed effect modelling procedure to assess evidence for an interaction between emotion (neutral or fearful faces) and expectation (expected or unexpected).

**FIGURE 2:**
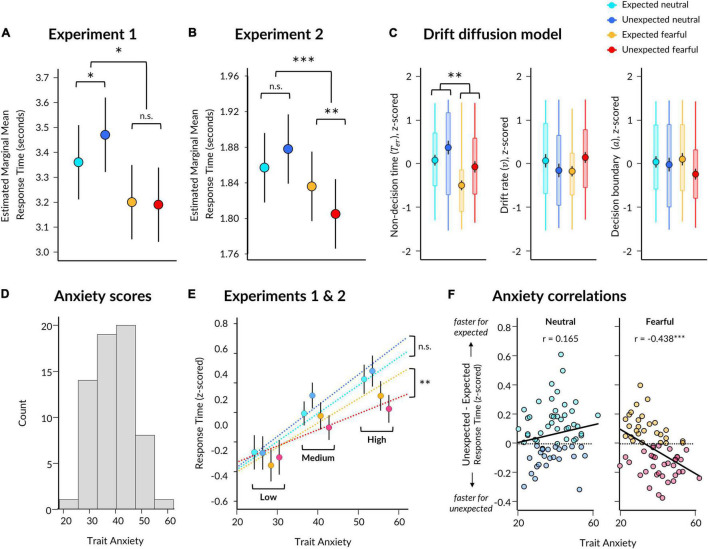
Behavioural results from Experiments 1 and 2. **(A)** Estimated marginal means for each condition from a linear mixed effects model for Experiment 1. Significant two-way interaction and simple effects are indicated by asterisks. Error bars represent standard error. **(B)** Same as **(A)** except for Experiment 2. **(C)** Parameter estimates per condition derived from the EZ Drift Diffusion model. Markers represent mean across all subjects, with black vertical lines indicating standard error of the mean. Boxes represent 25th and 75th percentiles and coloured vertical lines indicate range across subjects. Significant main effect of emotion is indicated for non-decision time. **(D)** Trait anxiety scores for participants from both Experiments 1 and 2. **(E)** Estimated slopes from the linear mixed effects model of response time, which included a three-way interaction between expectation, emotion, and trait anxiety (x axis). Participant mean z-scored response times are shown (markers with error bars indicating standard error of the mean) with participants grouped into low (*z* < −0.5), medium (*z* = −0.5 and = 0.5), and high (*z* > 0.5) trait anxiety for visualisation purposes only. **(F)** Correlations between trait anxiety (x axis) and the effect of expectation (unexpected minus expected; y axis) on z-scored response times to neutral (left) and fearful (right) faces, using pooled data from Experiments 1 and 2. Higher scores on the y axis indicate faster responses to expected faces (lighter points), while lower scores indicate faster responses to unexpected faces (darker points) for the respective facial expression. Pearson correlation coefficients are displayed. *p < 0.05, **p < 0.01, ***p < 0.001.

We discovered a significant effect of emotion (*p* = 0.212 × 10^–4^), where response times were 221 ms faster (SE = 26 ms) for fearful than neutral faces (Figure 2A and Table 1 for statistics). Critically, there was a significant interaction (*p* = 0.014), in which responses were 115 ms slower (SE = 37 ms) for unexpected than expected neutral faces (*p* = 0.010), while responses were 13 ms faster (SE = 37 ms) for unexpected than expected fearful faces (13 ms difference, SE = 37 ms, *p* = 0.985). Due to this interaction, the overall effect of expectation was not significant (*p* = 0.726, 51 ms difference, SE = 26 ms). Overall, these findings support the Survival Hypothesis, as fearful faces were rapidly detected regardless of prior expectations.

**TABLE 1:** Response time models for Experiments 1 and 2.

Experiment 1: RT ∼ Emotion × Expectation + (1 | Subject)				
**Fixed Effect**	**β**	**SE**	***t***	***P***
(Intercept)	3.198	0.151	21.120	< 2.00 × 10^–16^***
Emotion	0.157	0.037	4.254	2.12 × 10^–5^***
Expectation	–0.013	0.037	–0.351	0.726
Emotion × Expectation	0.129	0.052	2.460	0.014*

**Condition**	**EMM**	**SE**	**95% Lower CI**	**95% Upper CI**

Expected Neutral	3.355	0.149	3.063	3.647
Unexpected Neutral	3.471	0.149	3.179	3.763
Expected Fearful	3.198	0.149	2.906	3.490
Unexpected Fearful	3.185	0.149	2.893	3.477

**Experiment 2: RT ∼ Emotion × Expectation + (1 | Subject)**				

**Fixed Effect**	**β**	**SE**	***t***	***P***

(Intercept)	1.836	0.039	47.134	< 2.00 × 10^–16^***
Emotion	0.021	0.009	2.317	0.021*
Expectation	–0.031	0.009	–3.369	7.55 × 10^–4^***
Emotion × Expectation	0.051	0.013	3.980	6.93 × 10^–5^***

**Condition**	**EMM**	**SE**	**95% Lower CI**	**95% Upper CI**

Expected Neutral	1.857	0.039	1.781	1.933
Unexpected Neutral	1.878	0.039	1.801	1.954
Expected Fearful	1.836	0.039	1.759	1.912
Unexpected Fearful	1.805	0.039	1.729	1.881

The results from a linear mixed effects model of response time (formula displayed in top row). The top half reflects the results from Experiment 1 and the bottom half from Experiment 2. Within each half, the top section displays the statistics for each fixed effect (β = coefficient estimate, SE = standard error) and the bottom section displays the estimated marginal mean (EMM; in seconds) of each possible level of the fixed effects (CI = confidence interval). *p < 0.05, ***p < 0.001.

In Experiment 2, we aimed to replicate the findings from Experiment 1 and to also acquire measurements of neural activity using EEG while subjects performed the task. We repeated the same modelling procedure as in Experiment 1, which showed an effect of emotion (*p* = 0.021) where responses were 47 ms faster for fearful than neutral faces (SE = 6 ms; Figure 2B and Table 1). We also found a significant interaction between emotion and expectation (*p* = 0.693 × 10^–4^). For neutral faces, response times were 21 ms slower for unexpected than expected faces (*p* = 0.108), whereas for fearful faces, response times in Experiment 2 were 31 ms faster for unexpected than expected fearful faces (*p* = 0.004). Therefore, in this replication of Experiment 1, we again observed evidence for the Survival Hypothesis, such that response times were faster for fearful faces, even more so when they were unexpected.

#### Trait Anxiety Modulates Responses to Fearful Faces

Having observed faster responses to both expected and unexpected fearful faces, we pooled all data from both experiments (*N* = 63) to investigate whether individual differences in trait anxiety modulated the effects of emotion and expectation on response times. Due to the differences in trial lengths between the experiments, response times were first z-scored within each experiment. We constructed three models: (1) a null model, (2) an interaction model for emotion × expectation, and (3) an anxiety model, where trait anxiety (see Figure 2D for distribution) was added as a third interaction term (see section “Materials and Methods”). We discovered that the third model including anxiety explained response times above and beyond the emotion × expectation interaction model (BF_10_ = 2191, χ^2^ = 54.946, *p* = 3.336 × 10^–11^).

Subjects with higher trait anxiety were slower to respond overall (*p* = 0.009) but had a similar response time advantage to fearful faces compared to subjects with lower trait anxiety (anxiety × emotion interaction: *p* = 0.101; see Table 2 for full statistics). Crucially, however, subjects with higher trait anxiety exhibited a greater hastening of response times to unexpected fearful faces and slowing of response times to unexpected neutral faces compared to subjects with lower trait anxiety (anxiety × emotion × expectation interaction: *p* = 0.570 × 10^–3^; Figure 2E). Thus, the opposing effects of unexpected neutral and fearful faces on response time were driven predominantly by more anxious subjects.

**TABLE 2:** Response time models including anxiety for data pooled across Experiments 1 and 2.

Experiments 1 and 2: RT ∼ Emotion × Expectation × Trait Anxiety + Experiment + (1 | Subject)				
**Fixed effect**	**β**	**SE**	***t***	***P***

(Intercept)	0.005	0.096	0.048	0.961
Emotion	0.074	0.017	4.375	1.22 × 10^–5^***
Expectation	–0.049	0.017	–2.945	0.003***
Trait anxiety	0.019	0.007	2.692	0.009**
Experiment	–0.067	0.134	–0.502	0.618
Emotion × Expectation	0.111	0.024	4.663	3.14 × 10^–6^***
Emotion × Trait Anxiety	0.003	0.002	1.638	0.101
Expectation × Trait Anxiety	–0.006	0.002	–3.611	3.06 × 10^–4^***
Emotion × Expectation × Trait Anxiety	0.008	0.002	3.446	5.70 × 10^–4^***

**Simple slopes of Trait Anxiety**	**β**	**SE**	***t***	***P***

Expected neutral	0.021	0.007	3.103	0.003**
Unexpected neutral	0.024	0.007	3.423	0.001**
Expected fearful	0.019	0.007	2.693	0.009**
Unexpected fearful	0.012	0.007	1.786	0.079

The results from a linear mixed effects model of response time (formula displayed in top row). The top section displays the statistics for each fixed effect (β = coefficient estimate, SE = standard error) and the bottom section reports the estimated slope of each condition across trait anxiety, as displayed in Figure 2E. **p < 0.01, ***p < 0.001.

Using this pooled dataset, we investigated whether fearful faces were detected more quickly, irrespective of prior expectations (Experiment 1), or whether fearful faces were detected fastest when unexpected (Experiment 2). We averaged response times in each condition per participant and conducted Bayesian paired *t*-tests between expected and unexpected presentations of each facial expression. This revealed substantial evidence for there being slower response times to unexpected neutral (*M* = 0.102, SD = 0.596) than expected neutral (*M* = 0.037, SD = 0.570) faces (BF_10_ = 6.734), while the difference between unexpected fearful (*M* = −0.087, SD = 0.540) and expected fearful (*M* = −0.046, SD = 0.570) faces remained unclear (BF_10_ = 0.792).

We then correlated trait anxiety with each of these effects (unexpected minus expected, per facial expression), revealing that participants with higher trait anxiety were more likely to exhibit faster responses to unexpected than expected fearful faces than those with lower trait anxiety, who displayed the opposite (*r* = −0.438, *p* = 3.313 × 10^–4^, BF_10_ = 85.219; Figure 2F). The effect of expectation on response times to neutral faces did not significantly covary with trait anxiety (*r* = 0.165, *p* = 0.195, BF_10_ = 0.357). Therefore, these results support a *Survival Hypothesis* in which unexpected fearful faces are prioritised for conscious perception in those who are more anxious.

#### Response Times Reflect Non-decision Processes

While we observed an interaction between emotion and expectation across the two experiments, it was unclear the degree to which this interaction reflected a change in conscious breakthrough, versus a change in perceptual decision-making processes that occurred after a stimulus became consciously perceptible (e.g., conscious evidence accumulation). Although the orientation discrimination task was intended to be orthogonal to the experimental conditions, it is possible that participants adopted different strategies for perceptual decision-making between the two block types (e.g., adopting a different attentional set in neutral blocks than fearful blocks for specific visual features).

To tease apart the potential mechanisms underlying response time, we employed Drift Diffusion Modelling (Ratcliff, 1978); see section “Materials and Methods”) in which response times are modelled as a noisy increase in sensory evidence at a particular rate (*v*) from a starting point (*z*) to one of two possible decision boundaries (*a*). Here, decision boundaries represent a correct or incorrect decision in the orientation discrimination task. We reasoned that, if response times were predominantly driven by strategy-related differences in perceptual decision-making, then emotion and expectation would influence decision-related parameters (i.e., drift rate and decision boundary). Alternatively, if response times were predominantly driven by unconscious stimulus encoding, then emotion and expectation would influence a non-decision time parameter (*T*_*er*_), which encapsulates remaining processes such as sensory encoding and motor preparation. Here, we assume that motor preparation was equivalent across conditions, as suggested by pilot testing of a version of Experiment 1 where stimuli were presented normally on screen without interocular suppression (*N* = 30; mean response times, in seconds: expected neutral = 0.898, unexpected neutral = 0.905, expected fearful = 0.903, and unexpected fearful = 0.913; all models for effects of emotion and/or expectation were, at best, 0.4 less likely than a null model).

We selected a subset of participants (*N* = 39; 18 from Experiment 1, 21 from Experiment 2) who had sufficient numbers of error trials for drift diffusion modelling. We discovered significant effects of both emotion (*F*(37) = 11.242, *p* = 0.002) and expectation (*F*(37) = 5.925, *p* = 0.020) on non-decision time, such that non-decision time was shorter for fearful than neutral faces, and shorter for expected than unexpected faces (Figure 2C). There was no significant interaction (*F*(37) = 0.234, *p* = 0.631). A Bayesian repeated-measures ANOVA indicated that a model containing main effects of emotion and expectation best explained the data (BF_*M*_ = 8.521).

In contrast, there were no significant effects of emotion or expectation on parameters for drift rate (emotion: *F*(37) = 0.014, *p* = 0.907; expectation: *F*(37) = 0.008, *p* = 0.931; interaction: *F*(37) = 2.876, *p* = 0.098) or decision boundary (emotion: *F*(37) = 0.101, *p* = 0.753; expectation: *F*(37) = 1.761, *p* = 0.193; interaction: *F*(37) = 0.845, *p* = 0.364). Follow-up Bayesian testing indicated a null model best explained drift rate (BF_*M*_ = 9.405) and boundary (BF_*M*_ = 5.224) parameters. Therefore, these results suggest that response times were driven predominantly by perceptual processes related to unconscious stimulus encoding.

### Electroencephalography

In our behavioural analyses, we found that response times to neutral faces were delayed when neutral faces occurred unexpectedly, whereas response times to unexpected fearful faces were as fast (Experiment 1) or even faster (Experiment 2) than expected fearful faces, especially in participants with higher trait anxiety. Using the EEG data collected during the task, we sought to determine whether prior expectations also modulated the time course of underlying neural activity evoked by neutral and fearful faces during bCFS.

#### Central-Parietal Positivity Component

**FIGURE 3:**
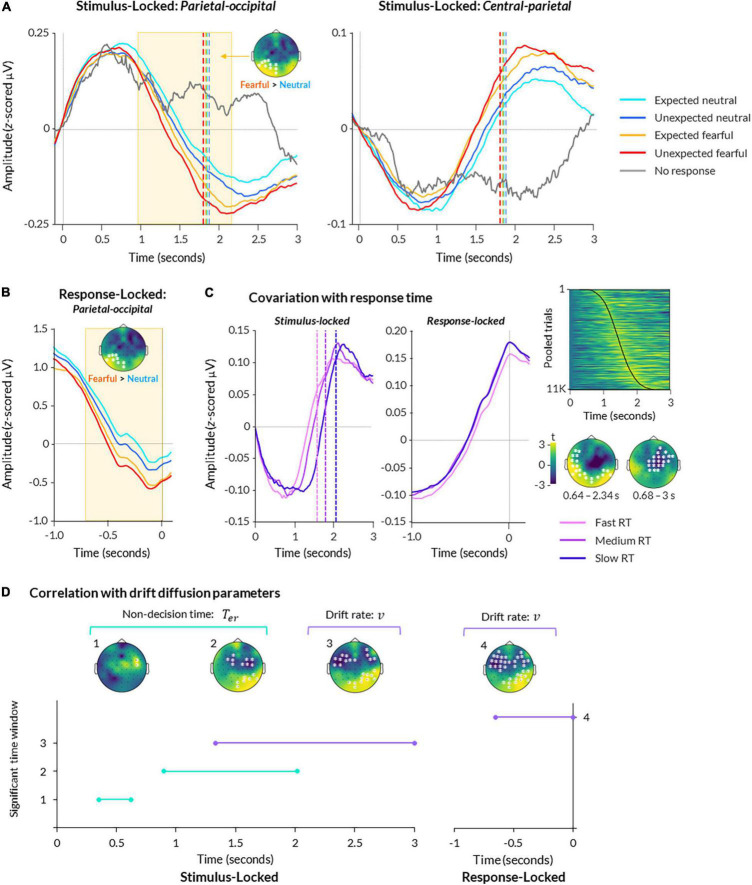
Electroencephalography (EEG) activity during bCFS. **(A)** ERPs are displayed for parietal-occipital (left) and central (right) electrodes. Data are averaged across all subjects and presented for each condition, plus trials where no response was made. Data are time-locked to trial onset. Significant time-periods and scalp topography from a cluster-based permutation test are indicated for the effect of emotion (fearful > neutral; *p* < 0.05). **(B)** Same as **(A)**, except data is response-locked data from parietal-occipital electrodes. **(C)** Data from central-parietal electrodes are shown for stimulus-locked (left) and response-locked (middle) epochs, with trials split into fast, medium, and slow response times (using 25th, 50th, and 75th percentiles of each subject’s data, respectively). A heatmap (upper right) displays central-parietal neural amplitude from all trials pooled across all participants, sorted in order of response time with fastest at the top and slowest at the bottom (response times indicated by black line; heatmap was smoothed with a 2 FWHM Gaussian kernel for visualisation). Scalp topographies (lower right) show two significant clusters of activity covarying with response time at the group level (*p* < 0.05). **(D)** A timeline of significant time windows for correlations between non-decision time (green lines) or drift rate (purple lines) and stimulus-locked (left graph) or response-locked (right graph) neural activity (*p* < 0.05, FDR-corrected). Significant time windows are denoted numbers (y axis) corresponding to the topographies shown above.

First, we examined neural activity time-locked to the onset of each trial, as well as the onset of response. Trials with incorrect responses and extreme response times were excluded (see section “Materials and Methods”). The average waveform across trials and participants revealed a sinusoidal pattern of activity, where neural amplitude peaked first at approximately 0.75 s (positive peak over parietal-occipital electrodes, negative peak over frontal-central electrodes) and then again with opposite polarity at approximately 2.25 s (Figure 3A). Due to there being no sudden stimulus onset, typical face-related ERP components such as a P100 and N170 were absent from neural activity, similar to previous studies using conscious suppression techniques (Sterzer et al., 2009; Kaunitz et al., 2011; Yokoyama et al., 2013; Schlossmacher et al., 2017; Engell and Quillian, 2020; Poland et al., 2021) and studies where visual stimuli gradually increase in contrast (O’Connell et al., 2012).

For comparison, trials in which participants failed to respond (39 trials on average across participants, ranging from 1 to 238) exhibited the first peak but not the second, suggesting the second peak related to perceptual decision-making and/or motor response (Figure 3A). Hence, the patterns of EEG activity closely resembled a decision-related central-parietal positivity (CPP) component, previously identified in studies on perceptual decision-making (O’Connell et al., 2012). This component encapsulates a slow increase in neural activity over central-parietal electrodes, peaking at the time of response (Figure 3B).

The CPP is commensurate with the spatial and temporal profile of the P300, a component typically observed as an increase in neural activity for deviants versus standards at approximately 300 ms post-trial onset over midline electrodes (Polich, 2007). Both the CPP and P300 are sensitive to the difficulty, timing, and accuracy of decision-making (O’Connell et al., 2012; Twomey et al., 2015; Nunez et al., 2019), as well as prior expectations (van den Brink et al., 2021), but see Rungratsameetaweemana et al., 2018b,a) and subjective perception of stimuli (Rutiku et al., 2015; Tagliabue et al., 2019). Thus, both components reflect the same neurological processes but differ in their temporal features, such that the P300 is typically time-locked to sudden stimulus onset (potentially making it more susceptible to distortion by sensory processing), while the CPP is locked to the formation of a perceptual decision itself (O’Connell et al., 2012).

To examine a statistical relationship between the CPP and responses, we performed a two-level cluster-based permutation analysis on all trials regressed against trial-by-trial response time. This revealed a significant relationship between neural activity and trial-by-trial response time, corresponding to two clusters: one spanning central and frontal electrodes from 0.680 to 3 s post-trial onset (*p* = 0.002), and another spanning occipital and temporal electrodes from 0.640 to 2.30 s post-trial onset (*p* = 0.002; Figure 3C).

#### Greater Neural Activity for Fearful Faces

We first sought to broadly determine when the time course of neural activity across the scalp could be distinguished between conditions. We performed a series of two-level cluster-based permutation tests for the effects of emotion, expectation, and their interaction. We examined EEG data from the full 3-second trial window (i.e., stimulus-locked; Figure 3A), as well as from a 1-second window preceding response onset (i.e., response-locked; Figure 3B; shortest observed response time across all subjects and all trials = 0.893 s).

For stimulus-locked data, we found a main effect of emotion, such that fearful faces elicited significantly greater amplitude overall than neutral faces (cluster spanning from 1.09 to 2.20 s post-trial onset across left occipital and parietal electrodes, *p* = 0.006; Figure 3A). This is in line with previous studies that report greater neural amplitude for fearful faces compared to neutral faces presented in paradigms that do not manipulate expectation (MacNamara et al., 2012; Bruchmann et al., 2020; Martin et al., 2021). There was no significant relationship with trait anxiety (all clusters *p* > 0.379).

Activity in response-locked epochs was also significantly greater for fearful than neutral faces, as reflected by a cluster from −0.71 to −0.02 s preceding response onset over left occipital and parietal electrodes (*p* = 0.004; Figure 3B). Together, these results suggest that fearful faces evoked significantly greater activity than neutral faces in both stimulus-locked and response-locked neural activity.

Next, we examined whether expectation broadly influenced spatiotemporal neural activity. Cluster-permutation testing across all electrodes and all time points did not return any significant effects of expectation (*p* > 0.262) or an emotion × expectation interaction (*p* > 0.266) across stimulus-locked epochs or response-locked epochs (for the latter, expectation: *p* > 0.999, interaction: *p* > 0.333). Thus, the effects of expectation and its interaction with emotion were observable at the behavioural level but not in broad patterns of spatiotemporal activity, similar to previous studies reporting non-significant effects of expectation in CPP amplitude (Rungratsameetaweemana et al., 2018b,a).

#### Non-decision Time and Drift Rate Covary With Neural Activity

We next investigated whether decision-making parameters of non-decision time, drift rate, and decision boundary covaried with spatiotemporal patterns of neural activity, as a means of mapping the temporal progression of different cognitive processes from trial start the conscious breakthrough. Within a subset of participants with sufficient error trials to conduct drift-diffusion modelling (*N* = 20), we conducted a group level analysis on the correlation between neural activity and drift diffusion parameters for each condition. For this analysis, we performed FDR correction instead of cluster-based permutation testing to allow us to make inferences about the timing of any observed effects whilst resolving issues with multiple comparisons (Fields and Kuperberg, 2020). Note that we only considered a time period significant if there were at least two neighbouring electrodes co-active at a time.

In stimulus-locked neural activity, we found that non-decision time significantly covaried with neural amplitude throughout two time periods: 0.35–0.62 s (frontal-central electrodes) and 0.90– 2.02 s (central, parietal, and occipital electrodes; *p* < 0.05 FDR-corrected; Figure 3D). In response-locked activity, we found no significant correlation between non-decision time and neural amplitude. Overall, these findings suggest two separable processes — one early (350–620 ms) and one late (900–2000 ms) — that likely relate to early and late sensory encoding rather than response-locked motor preparation.

For drift rate, we found a single time window of covariation from 1.33 to 3 s post-trial onset, over frontal-temporal-central and parietal-occipital electrodes (*p* < 0.05 FDR-corrected; Figure 3D). Similarly, we found a significant time period of covariation from -0.65 to 0 s pre-response onset over similar electrodes (*p* < 0.05 FDR-corrected). These results suggest that neural activity across the scalp increased as the rate of evidence accumulation increased in the lead up to response.

Finally, for decision boundary, we did not observe any significant brain-behaviour correlations in stimulus-locked or response-locked epochs (all *p* > 0.05, FDR-corrected).

#### Detecting Change Points in Neural Activity

The above findings indicate a temporal progression of neural processing from a period of initial sensory encoding approximately 350 ms after face stimuli began to fade in, followed by a transitionary period of sensory encoding and evidence accumulation in the lead up to breakthrough and subsequent perceptual decision-making. Thus, we narrowed our investigation to specifically examine whether prior expectations modulated these periods of sensory encoding and evidence accumulation differently for fearful than neutral faces. Similar approaches have been adopted by previous studies finding that the onset of the CPP reflects non-decision time and the slope reflects the rate of evidence accumulation (van Vugt et al., 2019; van den Brink et al., 2021).

**FIGURE 4:**
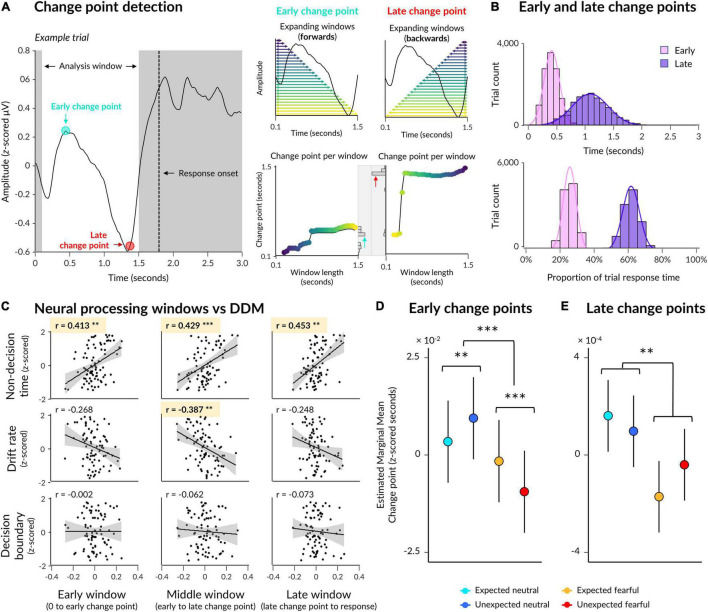
Early and late change points in neural activity. **(A)** Illustration of the change point detection procedure with an exemplar ERP from a single trial (left). An analysis window was defined 0.1 s post-trial onset to 0.3 s pre-response onset. Early change points were computed by detecting abrupt changes in signal in multiple expanding windows where most windows contained earlier time samples (upper middle: each window is indicated by a horizontal arrow, with longer windows indicated by lighter colours), and then taking the most frequent change point across all windows (lower middle: change points indicated on y axis for each window length on the x axis, with a histogram of change points across all windows displayed on the right). Late change points were detected in the same way, except with windows expanding from the end of the analysis window to the start (upper right) so that most windows contained later time samples. **(B)** Early and late change points are pooled across all trials from all subjects, with change points displayed in seconds (top) as well as a proportion of each trial’s response time (bottom). **(C)** Correlations between drift diffusion parameters (rows) and neural processing windows (columns). **(D)** Estimated marginal means from a multilevel model where emotion and expectation predicted early change point time. Significant interaction and simple effects are indicated by asterisks. **(E)** Same as **(D)** except for late change points. Asterisks indicate a significant main effect of emotion. *p < 0.05, ^**^p < 0.01, ^***^p < 0.001.

To map the time course of changes in neural processing, we detected abrupt changes in signal that occurred either towards the beginning of the trial or closer to response onset (see section “Materials and Methods” and Figure 4A). We then averaged these “early” and “late” change points across two clusters of electrodes: a bilateral parietal-occipital cluster (left: P7, P9, PO7; right: P8, P10, PO8), given the effect of emotion found via cluster-based permutation and our *a priori* hypotheses for visual encoding, and a central-parietal cluster (Cz, CPz, Cz), given the presence of a CPP component in our data.

On average across participants, an early change point occurred 0.473 s post-trial onset (SD = 0.063, range = 0.339–0.596) across all electrodes of interest (Figure 4B). Relative to trial-by-trial response time, this translates to 25.62% of a trial window from trial onset to response onset (SD = 0.33%, range = 24.91–26.31%). For comparison, a late change point occurred 1.137 s post-trial onset (SD = 0.159, range = 0.781–1.476), or 61.17% of each trial window (SD = 1.74%, range = 56.80–63.91%), on average. Thus, the average time between early and late change points was 0.665 s (SD = 0.107, range = 0.442–0.880), and the average time between late change points and response onset was 0.703 s (SD = 0.065, range = 0.565–0.835).

We investigated whether change points in neural activity mapped onto parameters of decision-making: namely, non-decision time, drift rate, and decision boundary. Using a subset of participants for which we had both EEG data and drift diffusion modelling data (*N* = 20, out of 31, with sufficient numbers of error trials), we correlated each drift rate parameter with three windows of neural processing: initial (time between trial onset and early change point), middle (time between early and late change point), and late (time between late change point and response onset). We z-scored data across conditions per participant, per data type (i.e., each window and each decision-making parameter), and performed correlations on the pooled dataset.

The above resulted in nine Pearson’s correlations, four of which were significant after applying a Bonferroni correction for multiple comparisons (Figure 4C). The duration of initial neural processing was significantly correlated with non-decision time (*r*(78) = 0.413, *p*_adj_ = 0.001) but not drift rate (*r*(78) = −0.267, *p*_adj_ = 0.147) or decision boundary (*r*(78) = −0.002, *p*_adj_ > 1), suggesting that this early time window relates to initial sensory encoding while face stimuli were very low contrast. The duration of the middle window, between early and late change points, was significantly correlated with both non-decision time (*r*(78) = 0.429, *p*_adj_ = 0.001) and drift rate (*r*(78) = −0.387, *p*_adj_ = 0.004) but not decision boundary (*r*(78) = −0.062, *p*_adj_ > 1), suggesting that this period captured both sensory encoding as well as evidence accumulation towards a decision in the orientation-discrimination task, where higher drift rates corresponded to shorter middle windows. Finally, the duration of the late window was significantly correlated only with nondecision time (*r*(78) = 0.453, *p*_adj_ = 2.245 × 10^–4^) but not with drift rate (*r*(78) = −0.248, *p*_adj_ = 0.242) nor with decision boundary (*r*(78) = −0.073, *p*_adj_ > 1), suggesting that this window relates to post-decision motor preparation.

#### Earlier Sensory Encoding of Unexpected Fearful Faces

Having identified three distinct time periods of neural processing related to sensory encoding, evidence accumulation, and decision execution, we investigated whether these periods were significantly influenced by prior expectations and emotional expression. We constructed two mixed effects models where change points (either early or late) were predicted by an interaction between emotion and expectation, with electrode cluster (parietal-occipital or central-parietal) and the proportion of response time added as fixed effects. For the model predicting late change points, we added additional fixed effects for the early change point and its proportion of response time to control for any dependency of late change points on early change points (correlation between time of early and late change points: *r* = 0.996, *p* < 2.200 × 10^–16^).

Our first model showed that early change points in neural activity were shifted earlier for fearful faces than neutral faces (β = 0.005, *p* = 0.004; see Table 3 for detailed statistics). Crucially, a significant interaction (β = 0.014, *p* = 9.189 × 10^–9^) revealed opposing effects of expectation on neutral and fearful faces, where initial change points occurred earlier for unexpected fearful faces than expected fearful faces (β = 0.008, *p* = 2.553 × 10^–5^), but earlier for expected neutral faces than unexpected neutral faces (β = −0.006, *p* = 0.002; Figure 4D). This suggests that the interaction we observed at the behavioural level was evident in neural activity as early as 470 ms post-trial onset, indicating that prior expectations influenced sensory encoding of consciously-suppressed stimuli. Thus, unexpected fearful faces were encoded more quickly and unexpected neutral faces were encoded more slowly than their expected counterparts.

**TABLE 3:** Change point models for Experiment 2.

Early: Change point ∼ Emotion × Expectation +				
Electrode group + RT proportion + (1 | Subject)				
**Fixed Effect**	**β**	**SE**	***t***	***p***

(Intercept)	−0.001	0.010	−0.136	0.892
Emotion	0.005	0.002	2.908	0.004**
Expectation	−0.008	0.002	−4.596	4.367 × 10^–6^***
Emotion × Expectation	0.014	0.002	5.747	9.189 × 10^–9^***
Electrode group	<0.001	0.001	−0.287	0.774
RT proportion	0.014	0.012	172.574	<2.00 × 10^–16^***

**Condition**	**EMM**	**SE**	**95% Lower CI**	**95% Upper CI**

Expected neutral	0.003	0.010	−0.017	0.024
Unexpected neutral	0.009	0.010	−0.011	0.030
Expected fearful	−0.002	0.010	−0.022	0.019
Unexpected fearful	−0.009	0.010	−0.030	0.011

**Late: Change point ∼ Emotion × Expectation + Electrode group + Late**				
**RT proportion + Early change point + Early RT proportion + (1 | Subject)**				

**Fixed effect**	**β**	**SE**	***t***	***P***

(Intercept)	−0.002	0.001	−1.440	0.156
Emotion	0.003	0.001	3.155	0.002**
Expectation	0.001	0.001	1.245	0.213
Emotion × Expectation	−0.002	0.001	−1.305	0.192
Electrode group	0.001	0.001	1.248	0.212
Late RT proportion	1.977	0.007	292.690	<2.00 × 10^–16^***
Early change point	2.240	0.004	523.077	<2.00 × 10^–16^***
Early RT proportion	−4.275	0.011	−382.496	<2.00 × 10^–16^***

**Condition**	**EMM**	**SE**	**95% Lower CI**	**95% Upper CI**

Expected neutral	1.857	0.039	1.781	1.933
Unexpected neutral	1.878	0.039	1.801	1.954
Expected fearful	1.836	0.039	1.759	1.912
Unexpected fearful	1.805	0.039	1.729	1.881

The results from two linear mixed effects models of neural change points (formula displayed in top row) for early (top half) and late (bottom half). The top section displays the statistics for each fixed effect (β = coefficient estimate, SE = standard error) and the bottom section displays the estimated marginal mean (EMM; in seconds) of each possible level of the fixed effects (CI = confidence interval). **p < 0.01, ***p < 0.001.

Our second model investigated whether late change points were also influenced by emotion and expectation, controlling for both response time and the previous (early) change point. The model showed that late change points occurred earlier when faces were fearful than when faces were neutral (β = 0.003, *p* = 0.002; Figure 4E). There was, however, no significant interaction with expectation (β = −0.002, *p* = 0.192). This suggests that the differential effect of prior expectations on response times to fearful vs neutral faces was likely driven by initial sensory encoding, while fearful faces increased neural amplitude and accelerated the time between encoding and decision-making.

## Discussion

The aim of this study was to explore how prediction errors for neutral and fearful faces influence perceptual decision-making under ambiguous viewing conditions. We presented neutral and fearful faces under interocular suppression (bCFS) in blocks with different presentation contingencies. We found that participants’ response times in a perceptual discrimination task were significantly faster for fearful faces, in line with previous studies reporting prioritised conscious access to emotional stimuli (Hedger et al., 2015; Gayet et al., 2016; Gomes et al., 2017). Response times were slower for unexpected neutral faces compared to expected neutral faces, similar to previous studies (Pinto et al., 2015; Meijs et al., 2018). Critically, however, the opposite was true for fearful faces, which evoked faster response times even when they were unexpected, an effect driven by participants with higher trait anxiety. By combining drift diffusion modelling with a change detection analysis of neural activity, we discovered that initial sensory encoding of fearful faces was accelerated when a fearful face was unexpected, while encoding of neutral faces was slower when unexpected. This novel finding supports our **Survival Hypothesis**, positing that prediction errors differentially influence the encoding of unconsciously-presented fearful and neutral faces.

Our findings shed light on the seemingly paradoxical theory behind how the brain constructs conscious visual percepts (Press et al., 2020). On the one hand, it is important that our perception is veridical. Hence, our perceptual experience is biased towards our prior expectations, as these constitute our best estimate of external reality (de Lange et al., 2018). On the other hand, it is important that our perception is informative. Thus, we might hypothesise that our perceptual experience is biased towards more surprising stimuli, as these contain critical information needed to update our current model of the world, particularly if the new information pertains to threat (Otten et al., 2017).

To reconcile this paradox, our findings support an opposing process model of expectation and conscious perception (Press et al., 2020). This model posits that neural representations of expected and unexpected stimuli are enhanced at different times throughout perceptual processing depending on the informative content. Initially, processing is biased towards our expectations (e.g., P100 response; Kouider et al., 2015; Yon and Press, 2017) but then is later enhanced for informative stimuli that violate our expectations (e.g., mismatch negativity response; Garrido et al., 2009). Here, converging evidence from a parameter-informed EEG analysis (in a subset of 20 participants, out of 31, with drift-diffusion modelling) and a trial-by-trial change point detection (with all 31 participants) suggested that initial sensory encoding was accelerated for neutral faces that matched prior expectations, thus hastening response times overall. Fearful faces, however, were encoded more rapidly when they violated expectations. This suggests that the informative value of fearful faces was rapidly detected at a stage when faces were very low contrast and thus unlikely to be consciously perceptible, in line with previous findings for affective processing of unconsciously-presented stimuli (Jiang et al., 2018).

Fearful faces are an innate threat cue for humans, and their characteristic low-level visual properties make them more perceptually salient (e.g., higher contrast due to exposed whites of the eyes; Hedger et al., 2015). Thus, fearful faces contain biologically-relevant information (even if not instrumental in the context of this task), as well as more precise perceptual information, relative to neutral faces. An anatomical pathway running from the superior colliculus to the amygdala via the pulvinar (effectively bypassing the visual cortex) has previously been shown to respond preferentially to fearful faces at very short latencies (Silverstein and Ingvar, 2015; Méndez-Bértolo et al., 2016), even when presented outside of awareness (Morris et al., 1999; Liddell et al., 2005; Ohman et al., 2007; Tamietto and de Gelder, 2010; McFadyen et al., 2019). This pathway is a candidate mechanism for the accelerated sensory encoding of fearful faces we observed in our data, a hypothesis that could be directly tested in future research.

Following expedited sensory encoding of unexpected fearful faces, our findings point towards there being an observable transitional period from unconscious to conscious perception as face stimuli gradually broke through into awareness. Correlations between neural activity and decision-making parameters revealed a temporal profile of neural processing in which an initial period of non-decision processing was followed by both non-decisional processes and an increase in evidence accumulation for whether faces were oriented left or right. We speculate that this window, corresponding to the time between “early” and “late” change points in pre-response neural activity, reflected ongoing sensory encoding of face stimuli alongside an accumulation of evidence for face orientation as faces became more consciously perceptible. Indeed, previous studies have found that neural activity increases to emotional stimuli more so when consciously perceptible (Hoffmann et al., 2012; Schlossmacher et al., 2017). Similarly, the CPP component (the primary component observed in our data) also scales with subjective stimulus visibility (Salti et al., 2012; Rutiku et al., 2015; Tagliabue et al., 2019). Hence, an increase in subjective perceptibility could explain the increased amplitude to fearful faces we observed during this interim window, although we remain sceptical due to challenges associated with inferring the contents of subjective experience (Sterzer et al., 2014).

Intriguingly, an interaction between emotional expression and prior expectations on response time was driven predominantly by subjects with higher trait anxiety. Previous research has shown that people with higher trait anxiety have heightened perceptual and attentional biases towards threat (Mogg et al., 2007; Grillon and Charney, 2011; Sussman et al., 2016; Damjanovic et al., 2017) even when unconsciously-presented (Duval et al., 2020). Unconscious attentional capture is a plausible explanation for the exaggerated response time advantage seen in more anxious participants for unexpected fearful faces, where more anxious participants might perceive fearful faces as being more informative than less anxious participants. Another explanation, however, is that less anxious participants are less susceptible to interocular suppression, thus producing a floor effect and diminishing the magnitude of any interaction between emotional expression and prior expectations on response time. To our knowledge, a relationship between trait anxiety and interocular suppression has not previously been reported, but may warrant future investigation.

It is important to note that, although the present study was motivated by previous research on affective stimuli and perceptual decision-making (Otten et al., 2017), we cannot ascertain the degree to which our findings reflect affective vs low-level visual processing due to the inherent visual differences between neutral and fearful faces (Hedger et al., 2015; Webb and Hibbard, 2020). As explained above, however, our findings present novel evidence for a two-process model of perception in which more surprising stimuli — whether due to their visual salience or their affective content — are prioritised for conscious access (Press et al., 2020).

Future research could disambiguate between affective and low-level visual effects by using stimuli with identical low-level visual properties but different affective associations, such as that achieved by fear conditioning (Gayet et al., 2016). This would also reduce any systematic differences in perceptual decision-making occurring after stimuli have broken through into conscious perception. For example, the task in the present study (i.e., reporting whether face stimuli were rotated clockwise or anticlockwise) might plausibly be made easier by less perceptually ambiguous, high-contrast eyes of fearful faces, thus resulting in response time differences relating to conscious rather than unconscious processing (Stein and Peelen, 2021).

Overall, our findings present novel evidence for a differential effect of prediction errors for fearful faces, as compared to neutral faces, on perceptual decision-making under highly ambiguous viewing conditions. These findings open many avenues for future research, including investigating the anatomical pathways that might subserve this differential effect, as well as exploring which aspects of stimuli (e.g., visual saliency, emotional content, task relevance, etc.) boost unconscious perceptual processing when prior expectations are violated.

## Data Availability Statement

The datasets presented in this study can be found in online repositories. The names of the repository/repositories and accession number(s) can be found below: https://osf.io/p3du5/.

## Ethics Statement

The studies involving human participants were reviewed and approved by University of Queensland’s Human Research Ethics Committee. The patients/participants provided their written informed consent to participate in this study.

## Author Contributions

JM, JBM, and MG ontributed to experimental design. JM, MG, and NT contributed to analysis design, with JM performing analyses. JM wrote the original manuscript. JM, JBM, NT, and MG contributed to editing. All authors contributed to the article and approved the submitted version.

## Conflict of Interest

The authors declare that the research was conducted in the absence of any commercial or financial relationships that could be construed as a potential conflict of interest.

## Publisher’s Note

All claims expressed in this article are solely those of the authors and do not necessarily represent those of their affiliated organizations, or those of the publisher, the editors and the reviewers. Any product that may be evaluated in this article, or claim that may be made by its manufacturer, is not guaranteed or endorsed by the publisher.

## References

[B1] Aizenberg M. Rolón-Martínez S. Pham T. Rao W. Haas J. S. Geffen M. N. (2019). Projection from the amygdala to the thalamic reticular nucleus amplifies cortical sound responses. *Cell Rep.* 29:1396. 10.1016/j.celrep.2019.10.036 31665648 PMC7094085

[B2] Aru J. Rutiku R. Wibral M. Singer W. Melloni L. (2016). Early effects of previous experience on conscious perception. *Neurosci. Consciousness* 2016:niw004. 10.1093/nc/niw004 30109125 PMC6084554

[B3] Barbosa L. S. Vlassova A. Kouider S. (2017). Prior expectations modulate unconscious evidence accumulation. *Consciousness Cogn.* 51 236–242. 10.1016/j.concog.2017.04.001 28411474

[B4] Bates D. Mächler M. Bolker B. Walker S. (2015). Fitting linear mixed-effects models usinglme4. *J. Statist. Software* 67:i01. 10.18637/jss.v067.i01

[B5] Beanland V. Tan C. H. Christensen B. K. (2018). The unexpected killer: effects of stimulus threat and negative affectivity on inattentional blindness. *Cogn. Emot.* 32 1374–1381. 10.1080/02699931.2017.1394269 29067866

[B6] BioSemi (2007). *Active Two 64 Ag-AgCl Electrode System.* Amsterdam: BioSemi.

[B7] Bruchmann M. Schindler S. Straube T. (2020). The spatial frequency spectrum of fearful faces modulates early and mid-latency ERPs but not the N170. *Psychophysiology* 57:e13597. 10.1111/psyp.13597 32390215

[B8] Calvillo D. P. Hawkins W. C. (2016). Animate objects are detected more frequently than inanimate objects in inattentional blindness tasks independently of threat. *J. General Psychol.* 143 101–115. 10.1080/00221309.2016.1163249 27055078

[B9] Chang A. Y. C. Kanai R. Seth A. K. (2015). Cross-modal prediction changes the timing of conscious access during the motion-induced blindness. *Consciousness Cogn.* 31 139–147. 10.1016/j.concog.2014.11.005 25486340

[B10] Chen C. Hu C.-H. Cheng Y. (2017). Mismatch negativity (MMN) stands at the crossroads between explicit and implicit emotional processing. *Hum. Brain Mapp.* 38 140–150. 10.1002/hbm.23349 27534834 PMC7133097

[B11] Chen Y. Li H. Jin Z. Shou T. Yu H. (2014). Feedback of the amygdala globally modulates visual response of primary visual cortex in the cat. *NeuroImage* 84 775–785. 10.1016/j.neuroimage.2013.09.010 24045078

[B12] Cornwell B. R. Garrido M. I. Overstreet C. Pine D. S. Grillon C. (2017). The unpredictive brain under threat: a neurocomputational account of anxious hypervigilance. *Biol. Psychiatry* 82 447–454. 10.1016/j.biopsych.2017.06.031 28838469 PMC5584579

[B13] Costello P. Jiang Y. Baartman B. McGlennen K. He S. (2009). Semantic and subword priming during binocular suppression. *Consciousness Cogn.* 18 375–382. 10.1016/j.concog.2009.02.003 19286396 PMC4521603

[B14] Damjanovic L. Meyer M. Sepulveda F. (2017). Raising the alarm: individual differences in the perceptual awareness of masked facial expressions. *Brain Cogn.* 114 1–10. 10.1016/j.bandc.2017.01.010 28258035

[B15] de Lange F. P. Heilbron M. Kok P. (2018). How do expectations shape perception? *Trends Cogn. Sci.* 22 764–779. 10.1016/j.tics.2018.06.002 30122170

[B16] Duval E. R. Lovelace C. T. Filion D. L. (2020). Attention allocation to subliminally presented affective faces in high and low social anxiety. *Int. J. Psychophysiol. Off. J. Int. Organiz. Psychophysiol.* 153 159–165. 10.1016/j.ijpsycho.2020.04.017 32389619

[B17] Engell A. D. Quillian H. M. (2020). Faces under continuous flash suppression capture attention faster than objects, but without a face-evoked steady-state visual potential: is curvilinearity responsible for the behavioral effect? *J. Vision* 20 14–14. 10.1167/jov.20.6.14 PMC7416886 38755795

[B18] Fields E. C. Kuperberg G. R. (2020). Having your cake and eating it too: flexibility and power with mass univariate statistics for ERP data. *Psychophysiology* 57:e13468. 10.1111/psyp.13468 31456213 PMC7269415

[B19] Fox J. Weisberg S. (2018). *An R Companion to Applied Regression.* Thousand Oaks, CA: SAGE Publications.

[B20] Friston K. Kiebel S. (2009). Predictive coding under the free-energy principle. *Philos. Trans. R. Soc. London Ser. Biol. Sci.* 364 1211–1221. 10.1098/rstb.2008.0300 19528002 PMC2666703

[B21] Gandolfo M. Downing P. E. (2019). Causal evidence for expression of perceptual expectations in category-selective extrastriate regions. *Curr. Biol.* 249:e3. 10.1016/j.cub.2019.06.024 31327721

[B22] Gao H. Jia Z. (2017). Detection of threats under inattentional blindness and perceptual load. *Curr. Psychol.* 36 733–739. 10.1007/s12144-016-9460-0

[B23] Garrido M. I. Kilner J. M. Stephan K. E. Friston K. J. (2009). The mismatch negativity: a review of underlying mechanisms. *Clin. Neurophysiol. Offi. J. Int. Federat. Clin. Neurophysiol.* 120 453–463. 10.1016/j.clinph.2008.11.029 19181570 PMC2671031

[B24] Gayet S. Paffen C. L. E. Belopolsky A. V. Theeuwes J. Van der Stigchel S. (2016). Visual input signaling threat gains preferential access to awareness in a breaking continuous flash suppression paradigm. *Cognition* 149 77–83. 10.1016/j.cognition.2016.01.009 26807500

[B25] Gomes N. Silva S. Silva C. F. Soares S. C. (2017). Beware the serpent: the advantage of ecologically-relevant stimuli in accessing visual awareness. *Evolu. Hum. Behav. Offi. J. Hum. Behav. Evolu. Soc.* 38 227–234. 10.1016/j.evolhumbehav.2016.10.004

[B26] Gordon N. Koenig-Robert R. Tsuchiya N. van Boxtel J. J. Hohwy J. (2017). Neural markers of predictive coding under perceptual uncertainty revealed with hierarchical frequency tagging. *eLife* 6:22749. 10.7554/eLife.22749 28244874 PMC5360443

[B27] Grillon C. Charney D. R. (2011). In the face of fear: anxiety sensitizes defensive responses to fearful faces. *Psychophysiology* 48 1745–1752. 10.1111/j.1469-8986.2011.01268.x 21824155 PMC3212615

[B28] Hedger N. Adams W. J. Garner M. (2015). Fearful faces have a sensory advantage in the competition for awareness. *J. Exp. Psychol. Hum. Percept. Performance* 41 1748–1757. 10.1037/xhp0000127 26280260

[B29] Hesselmann G. Sadaghiani S. Friston K. J. Kleinschmidt A. (2010). Predictive coding or evidence accumulation? False inference neuronal fluctuations. *PLoS One* 5:e9926. 10.1371/journal.pone.0009926 20369004 PMC2848028

[B30] Hoffmann M. Lipka J. Mothes-Lasch M. Miltner W. H. R. Straube T. (2012). Awareness modulates responses of the amygdala and the visual cortex to highly arousing visual threat. *NeuroImage* 62 1439–1444. 10.1016/j.neuroimage.2012.05.064 22659485

[B31] Hohwy J. (2012). Attention and conscious perception in the hypothesis testing brain. *Front. Psychol.* 3:96. 10.3389/fpsyg.2012.00096 22485102 PMC3317264

[B32] Hohwy J. Roepstorff A. Friston K. (2008). Predictive coding explains binocular rivalry: an epistemological review. *Cognition* 108 687–701. 10.1016/j.cognition.2008.05.010 18649876

[B33] Imbriano G. Sussman T. J. Jin J. Mohanty A. (2020). The role of imagery in threat-related perceptual decision making. *Emotion* 20 1495–1501. 10.1037/emo0000610 31192666 PMC6908763

[B34] JASP Team (2020). *JASP (version 0.11.1)[Computer Software].*

[B35] Jiang Y. Costello P. He S. (2007). Processing of invisible stimuli: advantage of upright faces and recognizable words in overcoming interocular suppression. *Psychol. Sci.* 18 349–355. 10.1111/j.1467-9280.2007.01902.x 17470261

[B36] Jiang Y. Wu X. Saab R. Xiao Y. Gao X. (2018). Time course of influence on the allocation of attentional resources caused by unconscious fearful faces. *Neuropsychologia* 113 104–110. 10.1016/j.neuropsychologia.2018.04.001 29626497

[B37] Kaunitz L. N. Kamienkowski J. E. Olivetti E. Murphy B. Avesani P. Melcher D. P. (2011). Intercepting the first pass: rapid categorization is suppressed for unseen stimuli. *Front. Psychol.* 2:198. 10.3389/fpsyg.2011.00198 21897827 PMC3160141

[B38] Kiebel S. J. Daunizeau J. Friston K. J. (2008). A hierarchy of time-scales and the brain. *PLoS Comput. Biol.* 4:e1000209. 10.1371/journal.pcbi.1000209 19008936 PMC2568860

[B39] Killick R. Fearnhead P. Eckley I. A. (2012). Optimal detection of changepoints with a linear computational cost. *J. Am. Statist. Assoc.* 107 1590–1598. 10.1007/s11222-016-9687-5 32063685 PMC6994226

[B40] Kok P. Brouwer G. J. van Gerven M. A. J. de Lange F. P. (2013). Prior expectations bias sensory representations in visual cortex. *J. Neurosci. Offi. J. Soc. Neurosci.* 33 16275–16284. 10.1523/JNEUROSCI.0742-13.2013 24107959 PMC6618350

[B41] Kok P. Jehee J. F. M. de Lange F. P. (2012). Less is more: expectation sharpens representations in the primary visual cortex. *Neuron* 75 265–270. 10.1016/j.neuron.2012.04.034 22841311

[B42] Kok P. Mostert P. de Lange F. P. (2017). Prior expectations induce prestimulus sensory templates. *Proc. Natl. Acad. Sci. U.S.A.* 114 10473–10478. 10.1073/pnas.1705652114 28900010 PMC5625909

[B43] Kouider S. Long B. Le Stanc L. Charron S. Fievet A.-C. Barbosa L. S. (2015). Neural dynamics of prediction and surprise in infants. *Nat. Commun.* 6:8537. 10.1038/ncomms9537 26460901 PMC4633815

[B44] Kovarski K. Latinus M. Charpentier J. Cléry H. Roux S. Houy-Durand E. (2017). Facial expression related vMMN: disentangling emotional from neutral change detection. *Front. Hum. Neurosci.* 11:18. 10.3389/fnhum.2017.00018 28194102 PMC5277013

[B45] Kuznetsova A. Brockhoff P. B. Christensen R. H. B. (2017). lmerTest package: tests in linear mixed effects models. *J. Statist. Software* 82:i13. 10.18637/jss.v082.i13

[B46] Lavielle M. (2005). Using penalized contrasts for the change-point problem. *Signal Proc.* 85 1501–1510. 10.1002/bimj.201700196 30475389

[B47] Liddell B. J. Brown K. J. Kemp A. H. Barton M. J. Das P. Peduto A. (2005). A direct brainstem-amygdala-cortical “alarm” system for subliminal signals of fear. *NeuroImage* 24 235–243. 10.1016/j.neuroimage.2004.08.016 15588615

[B48] Lieder F. Daunizeau J. Garrido M. I. Friston K. J. Stephan K. E. (2013). Modelling trial-by-trial changes in the mismatch negativity. *PLoS Comput. Biol.* 9:e1002911. 10.1371/journal.pcbi.1002911 23436989 PMC3578779

[B49] Lufityanto G. Donkin C. Pearson J. (2016). Measuring intuition: nonconscious emotional information boosts decision accuracy and confidence. *Psychol. Sci.* 27 622–634. 10.1177/0956797616629403 27052557

[B50] Lundqvist D. Flykt A. Öhman A. (1998). *Karolinska Directed Emotional Faces.* Stockholm: Department of Clinical Neuroscience, Psychology section, Karolinska Institutet.

[B51] MacNamara A. Schmidt J. Zelinsky G. J. Hajcak G. (2012). Electrocortical and ocular indices of attention to fearful and neutral faces presented under high and low working memory load. *Biol. Psychol.* 91 349–356. 10.1016/j.biopsycho.2012.08.005 22951516

[B52] Maris E. Oostenveld R. (2007). Nonparametric statistical testing of EEG- and MEG-data. *J. Neurosci. Methods* 164 177–190. 10.1016/j.jneumeth.2007.03.024 17517438

[B53] Martin A. Becker S. I. Pegna A. J. (2021). Attention is prioritised for proximate and approaching fearful faces. *Cortex J. Dev. Study Nervous Syst. Behav.* 134 52–64. 10.1016/j.cortex.2020.10.011 33249300

[B54] McFadyen J. Mattingley J. B. Garrido M. I. (2019). An afferent white matter pathway from the pulvinar to the amygdala facilitates fear recognition. *eLife* 8:40766. 10.7554/eLife.40766 30648533 PMC6335057

[B55] Meijs E. L. Slagter H. A. de Lange F. P. van Gaal S. (2018). Dynamic interactions between top–down expectations and conscious awareness. *J. Neurosci. Offi. J. Soc. Neurosci.* 38 2318–2327. 10.1523/JNEUROSCI.1952-17.2017 29386259 PMC6596276

[B56] Méndez-Bértolo C. Moratti S. Toledano R. Lopez-Sosa F. Martínez-Alvarez R. Mah Y. H. (2016). A fast pathway for fear in human amygdala. *Nat. Neurosci.* 19 1041–1049. 10.1038/nn.4324 27294508

[B57] Miles W. R. (1930). Ocular dominance in human adults. *J. Gen. Psychol.* 3 412–430.

[B58] Mogg K. Garner M. Bradley B. P. (2007). Anxiety and orienting of gaze to angry and fearful faces. *Biol. Psychol.* 76 163–169. 10.1016/j.biopsycho.2007.07.005 17764810 PMC2075532

[B59] Morris J. S. Öhman A. Dolan R. J. (1999). A subcortical pathway to the right amygdala mediating “unseen” fear. *Proc. Natl. Acad. Sci. U.S.A.* 96 1680–1685. 10.1073/pnas.96.4.1680 9990084 PMC15559

[B60] New J. J. Joshua J. German T. C. (2015). Spiders at the cocktail party: an ancestral threat that surmounts inattentional blindness. *Evolu. Hum. Behav.* 36 165–173. 10.1016/j.evolhumbehav.2014.08.004

[B61] Nunez M. D. Gosai A. Vandekerckhove J. Srinivasan R. (2019). The latency of a visual evoked potential tracks the onset of decision making. *NeuroImage* 197 93–108. 10.1016/j.neuroimage.2019.04.052 31028925

[B62] O’Connell R. G. Dockree P. M. Kelly S. P. (2012). A supramodal accumulation-to-bound signal that determines perceptual decisions in humans. *Nat. Neurosci.* 15 1729–1735. 10.1038/nn.3248 23103963

[B63] Ohman A. (2005). The role of the amygdala in human fear: automatic detection of threat. *Psychoneuroendocrinology* 30 953–958. 10.1016/j.psyneuen.2005.03.019 15963650

[B64] Ohman A. Carlsson K. Lundqvist D. Ingvar M. (2007). On the unconscious subcortical origin of human fear. *Physiol. Behav.* 92 180–185. 10.1016/j.physbeh.2007.05.057 17599366

[B65] Olszanowski M. Pochwatko G. Kuklinski K. Scibor-Rylski M. Lewinski P. Ohme R. K. (2015). Warsaw set of emotional facial expression pictures: a validation study of facial display photographs. *Front. Psychol.* 5:1516. 10.3389/fpsyg.2014.01516 25601846 PMC4283518

[B66] Oostenveld R. Fries P. Maris E. Schoffelen J.-M. (2011). FieldTrip: open source software for advanced analysis of MEG, EEG, and invasive electrophysiological data. *Comput. Intelli. Neurosci.* 2011:156869. 10.1155/2011/156869 21253357 PMC3021840

[B67] Otten M. Seth A. K. Pinto Y. (2017). A social bayesian brain: how social knowledge can shape visual perception. *Brain Cogn.* 112 69–77. 10.1016/j.bandc.2016.05.002 27221986

[B68] Ouden H. E. M. den Ouden H. E. M. Kok P. de Lange F. P. (2012). How prediction errors shape perception, attention, and motivation. *Front. Psychol.* 3:548. 10.3389/fpsyg.2012.00548 23248610 PMC3518876

[B69] Panichello M. F. Cheung O. S. Bar M. (2012). Predictive feedback and conscious visual experience. *Front. Psychol.* 3:620. 10.3389/fpsyg.2012.00620 23346068 PMC3549576

[B70] Parasuraman R. Galster S. (2013). Sensing, assessing, and augmenting threat detection: behavioral, neuroimaging, and brain stimulation evidence for the critical role of attention. *Front. Hum. Neurosci.* 7:273. 10.3389/fnhum.2013.00273 23781194 PMC3679495

[B71] Pinto Y. van Gaal S. de Lange F. P. Lamme V. A. F. Seth A. K. (2015). Expectations accelerate entry of visual stimuli into awareness. *J. Vision* 15:13. 10.1167/15.8.13 26114676

[B72] Poland E. Bhonsle A. Steinmann I. Wilke M. (2021). Reduced alpha amplitudes predict perceptual suppression. *Scientific Reports* 11 13040. 10.1038/s41598-021-92404-8 34158567 PMC8219776

[B73] Polich J. (2007). Updating P300: an integrative theory of P3a and P3b. *Clin. Neurophysiol. Offi. J. Int. Federat. Clin. Neurophysiol.* 118 2128–2148. 10.1016/j.clinph.2007.04.019 17573239 PMC2715154

[B74] Prins N. Kingdom F. A. A. (2009). *Palamedes: Matlab Routines for Analyzing Psychophysical Data [Computer software].*

[B75] Press C. Kok P. Yon D. (2020). The perceptual prediction paradox. *Trends Cogn. Sci.* 24 13–24. 10.1016/j.tics.2019.11.003 31787500

[B76] Rao R. P. Ballard D. H. (1999). Predictive coding in the visual cortex: a functional interpretation of some extra-classical receptive-field effects. *Nat. Neurosci.* 2 79–87. 10.1038/4580 10195184

[B77] Ratcliff R. (1978). A theory of memory retrieval. *Psychol. Rev.* 85 59–108. 10.1037/0033-295x.85.2.59

[B78] Rungratsameetaweemana N. Itthipuripat S. Salazar A. Serences J. (2018a). Expectations about low-level visual features influence late stages of cortical information processing. *J. Vision* 18:1051. 10.1167/18.10.1051

[B79] Rungratsameetaweemana N. Itthipuripat S. Salazar A. Serences J. T. (2018b). Expectations do not alter early sensory processing during perceptual decision-making. *J. Neurosci. Offi. J. Soc. Neurosci.* 38 5632–5648. 10.1523/JNEUROSCI.3638-17.2018 29773755 PMC8174137

[B80] Rutiku R. Martin M. Bachmann T. Aru J. (2015). Does the P300 reflect conscious perception or its consequences? *Neuroscience* 298 180–189. 10.1016/j.neuroscience.2015.04.029 25907442

[B81] Salti M. Bar-Haim Y. Lamy D. (2012). The P3 component of the ERP reflects conscious perception, not confidence. *Consciousness Cogn.* 21 961–968. 10.1016/j.concog.2012.01.012 22341937

[B82] Sassenhagen J. Draschkow D. (2019). Cluster-based permutation tests of MEG/EEG data do not establish significance of effect latency or location. *Psychophysiology* 56:e13335. 10.1111/psyp.13335 30657176

[B83] Schlossmacher I. Junghöfer M. Straube T. Bruchmann M. (2017). No differential effects to facial expressions under continuous flash suppression: an event-related potentials study. *NeuroImage* 163 276–285. 10.1016/j.neuroimage.2017.09.034 28939431

[B84] Schmack K. Burk J. Haynes J.-D. Sterzer P. (2016). Predicting subjective affective salience from cortical responses to invisible object stimuli. *Cerebral Cortex* 26 3453–3460. 10.1093/cercor/bhv174 26232987

[B85] Silverstein D. N. Ingvar M. (2015). A multi-pathway hypothesis for human visual fear signaling. *Front. Syst. Neurosci.* 9:101. 10.3389/fnsys.2015.00101 26379513 PMC4547041

[B86] Singer N. Eapen M. Grillon C. Ungerleider L. G. Hendler T. (2012). Through the eyes of anxiety: dissecting threat bias via emotional-binocular rivalry. *Emotion* 12 960–969. 10.1037/a0027070 22390706 PMC3468657

[B87] Smith R. Lane R. D. (2016). Unconscious emotion: a cognitive neuroscientific perspective. *Neurosci. Biobehav. Rev.* 69 216–238. 10.1016/j.neubiorev.2016.08.013 27522011

[B88] Spielberger C. D. Gonzalez-Reigosa F. Martinez-Urrutia A. Natalicio L. F. Natalicio D. S. (1971). The state-trait anxiety inventory. *Interam. J. Psychol.* 5 145–158.

[B89] Stein T. Peelen M. V. (2021). Dissociating conscious and unconscious influences on visual detection effects. *Nat. Hum. Behav.* 5 612–624. 10.1038/s41562-020-01004-5 33398144

[B90] Stein T. Sterzer P. Peelen M. V. (2012). Privileged detection of conspecifics: evidence from inversion effects during continuous flash suppression. *Cognition* 125 64–79. 10.1016/j.cognition.2012.06.005 22776239

[B91] Sterzer P. Jalkanen L. Rees G. (2009). Electromagnetic responses to invisible face stimuli during binocular suppression. *NeuroImage* 46 803–808. 10.1016/j.neuroimage.2009.02.046 19285140

[B92] Stein T. Seymour K. Hebart M. N. Sterzer P. (2014). Rapid fear detection relies on high spatial frequencies. *Psychol. Sci.* 25 566–574. 24379157 10.1177/0956797613512509

[B93] Sterzer P. Stein T. Ludwig K. Rothkirch M. Hesselmann G. (2014). Neural processing of visual information under interocular suppression: a critical review. *Front. Psychol.* 5:453. 10.3389/fpsyg.2014.00453 24904469 PMC4032950

[B94] Straube T. Mentzel H.-J. Miltner W. H. R. (2006). Neural mechanisms of automatic and direct processing of phobogenic stimuli in specific phobia. *Biol. Psychiatry* 59 162–170. 10.1016/j.biopsych.2005.06.013 16139812

[B95] Summerfield C. de Lange F. P. (2014). Erratum: expectation in perceptual decision making: neural and computational mechanisms. *Nat. Rev. Neurosci.* 15 816–816. 10.1038/nrn3863 25315388

[B96] Sussman T. J. Szekely A. Hajcak G. Mohanty A. (2016). It’s all in the anticipation: how perception of threat is enhanced in anxiety. *Emotion* 16 320–327. 10.1037/emo0000098 26479770

[B97] Sussman T. J. Weinberg A. Szekely A. Hajcak G. Mohanty A. (2017). Here comes trouble: prestimulus brain activity predicts enhanced perception of threat. *Cerebral Cortex* 27 2695–2707. 10.1093/cercor/bhw104 27114179

[B98] Tagliabue C. F. Veniero D. Benwell C. S. Y. Cecere R. Savazzi S. Thut G. (2019). The EEG signature of sensory evidence accumulation during decision formation closely tracks subjective perceptual experience. *Sci. Rep.* 9:4949. 10.1038/s41598-019-41024-4 30894558 PMC6426990

[B99] Tamietto M. de Gelder B. (2010). Neural bases of the non-conscious perception of emotional signals. *Nat. Rev. Neurosci.* 11 697–709. 10.1038/nrn2889 20811475

[B100] Tipples J. (2015). Rapid temporal accumulation in spider fear: evidence from hierarchical drift diffusion modelling. *Emotion* 15 742–751. 10.1037/emo0000079 25938616

[B101] Tottenham N. Tanaka J. W. Leon A. C. McCarry T. Nurse M. Hare T. A. (2009). The NimStim set of facial expressions: judgments from untrained research participants. *Psychiatry Res.* 168 242–249. 19564050 10.1016/j.psychres.2008.05.006 PMC3474329

[B102] Tsuchiya N. Koch C. (2005). Continuous flash suppression reduces negative afterimages. *Nat. Neurosci.* 8 1096–1101. 10.1038/nn1500 15995700

[B103] Twomey D. M. Murphy P. R. Kelly S. P. O’Connell R. G. (2015). The classic P300 encodes a build-to-threshold decision variable. *Eur. J. Neurosci.* 42 1636–1643. 10.1111/ejn.12936 25925534

[B104] van den Brink R. L. Murphy P. R. Desender K. de Ru N. Nieuwenhuis S. (2021). Temporal expectation hastens decision onset but does not affect evidence quality. *J. Neurosci. Offi. J. Soc. Neurosci.* 41 130–143. 10.1523/JNEUROSCI.1103-20.2020 33172980 PMC7786203

[B105] Van der Schalk J. Hawk S. T. Fischer A. H. Doosje B. (2011). Moving faces, looking places: validation of the Amsterdam dynamic facial expression set (ADFES). *Emotion* 11 907–920. 10.1037/a0023853 21859206

[B106] van Ravenzwaaij D. Donkin C. Vandekerckhove J. (2017). The EZ diffusion model provides a powerful test of simple empirical effects. *Psychonomic Bull. Rev.* 24 547–556. 10.3758/s13423-016-1081-y 27352898 PMC5389995

[B107] van Vugt M. K. Beulen M. A. Taatgen N. A. (2019). Relation between centro-parietal positivity and diffusion model parameters in both perceptual and memory-based decision making. *Brain Res.* 1715 1–12. 10.1016/j.brainres.2019.03.008 30876858

[B108] Vetter P. Sanders L. L. O. Muckli L. (2014). Dissociation of prediction from conscious perception. *Perception* 43 1107–1113. 10.1068/p7766 25509687

[B109] Vieira J. B. Wen S. Oliver L. D. Mitchell D. G. V. (2017). Enhanced conscious processing and blindsight-like detection of fear-conditioned stimuli under continuous flash suppression. *Exp. Brain Res. Exp. Hirnforschung Exp. Cerebrale* 235 3333–3344. 10.1007/s00221-017-5064-7 28815269

[B110] Vogel B. O. Shen C. Neuhaus A. H. (2015). Emotional context facilitates cortical prediction error responses. *Hum. Brain Mapp.* 36 3641–3652. 10.1002/hbm.22868 26047176 PMC6869547

[B111] Wagenmakers E.-J. van der Maas H. L. J. Grasman R. P. P. P. (2007). An EZ-diffusion model for response time and accuracy. *Psychonomic Bull. Rev.* 14 3–22. 10.3758/bf03194023 17546727

[B112] Webb A. L. M. Hibbard P. B. (2020). Suppression durations for facial expressions under breaking continuous flash suppression: effects of faces’ low-level image properties. *Sci. Rep.* 10:17427. 10.1038/s41598-020-74369-2 33060699 PMC7567108

[B113] Wiemer J. Gerdes A. B. M. Pauli P. (2013). The effects of an unexpected spider stimulus on skin conductance responses and eye movements: an inattentional blindness study. *Psychol. Res.* 77 155–166. 10.1007/s00426-011-0407-7 22227916

[B114] Willenbockel V. Sadr J. Fiset D. Horne G. O. Gosselin F. Tanaka J. W. (2010). Controlling low-level image properties: the SHINE toolbox. *Behav. Res. Methods* 42 671–684. 20805589 10.3758/BRM.42.3.671

[B115] Yokoyama T. Noguchi Y. Kita S. (2013). Unconscious processing of direct gaze: evidence from an ERP study. *Neuropsychologia* 51 1161–1168. 10.1016/j.neuropsychologia.2013.04.002 23603242

[B116] Yon D. Press C. (2017). Predicted action consequences are perceptually facilitated before cancellation. *J. Exp. Psychol. Hum. Percept. Perform.* 43 1073–1083. 10.1037/xhp0000385 28263639

